# Clinical and parasitological factors in parasite persistence after treatment and clinical cure of cutaneous leishmaniasis

**DOI:** 10.1371/journal.pntd.0005713

**Published:** 2017-07-13

**Authors:** Alvaro J. Martínez-Valencia, Carlos Frisherald Daza-Rivera, Mariana Rosales-Chilama, Alexandra Cossio, Elkin J. Casadiego Rincón, Mayur M. Desai, Nancy Gore Saravia, María Adelaida Gómez

**Affiliations:** 1 Centro Internacional de Entrenamiento e Investigaciones Médicas-CIDEIM, Cali, Colombia; 2 Hospital Universitario Centro Dermatológico Federico Lleras Acosta, Bogotá, Colombia; 3 Yale School of Public Health, Department of Chronic Disease Epidemiology, New Haven, Connecticut, United States of America; Instituto de Ciências Biológicas, Universidade Federal de Minas Gerais, BRAZIL

## Abstract

**Background:**

The determinants of parasite persistence or elimination after treatment and clinical resolution of cutaneous leishmaniasis (CL) are unknown. We investigated clinical and parasitological parameters associated with the presence and viability of *Leishmania* after treatment and resolution of CL caused by *L*. *Viannia*.

**Methods:**

Seventy patients who were treated with meglumine antimoniate (n = 38) or miltefosine (n = 32) and cured, were included in this study. *Leishmania* persistence and viability were determined by detection of kDNA and 7SLRNA transcripts, respectively, before, at the end of treatment (EoT), and 13 weeks after initiation of treatment in lesions and swabs of nasal and tonsillar mucosa.

**Results:**

Sixty percent of patients (42/70) had evidence of *Leishmania* persistence at EoT and 30% (9/30) 13 weeks after treatment initiation. A previous episode of CL was found to be a protective factor for detectable *Leishmania* persistence (OR: 0.16, 95%CI: 0.03–0.92). kDNA genotyping could not discern differences between parasite populations that persisted and those isolated at diagnosis.

**Conclusions:**

*Leishmania* persist in skin and mucosal tissues in a high proportion of patients who achieved therapeutic cure of CL. This finding prompts assessment of the contribution of persistent infection in transmission and endemicity of CL, and in disease reactivation and protective immunity.

## Author summary

Control of cutaneous leishmaniasis (CL) in the Americas is dependent upon active case detection and treatment. The efficacy and effectiveness of therapeutic interventions is based on clinical resolution of disease, not on parasitological clearance. The detection of dermotropic *Leishmania* in tissues such as nasal and conjunctival mucosa, blood and healthy skin in the absence of signs and symptoms of disease, suggests that despite clinical resolution, parasites persist subclinically. We examined clinical and parasitological factors associated with *Leishmania* persistence after standard-of-care treatment of CL caused by *L*. *Viannia*. We found that a high proportion of CL patients with therapeutically achieved clinical resolution of CL harbor viable *Leishmania*. A previous episode of CL was found to be a protective factor for parasite persistence. Treated, clinically cured CL patients constitute an important proportion of a persistently infected human population whose clinical and epidemiological significance remains to be determined.

## Introduction

Over 95% of clinical manifestations of human infections caused by *Leishmania* species of the *Viannia* subgenus consist of cutaneous lesions. Although *L*. *Viannia* species are typically considered dermotropic, infection is systemic [1, 2]. The presence and viability of parasites in lesion scars and in otherwise healthy tissues, including blood, skin, nasal and conjunctival mucosa, have been documented in patients with active cutaneous disease [3–6] and in individuals with asymptomatic infection residing in endemic areas of *L*. *Viannia* transmission [7]. The ability of *L*. *Viannia* parasites to colonize host tissues without causing signs or symptoms of disease reflects a host-pathogen relationship that is permissive for microbial persistence.

Loss of susceptibility for meglumine antimoniate and miltefosine has been reported after a single treatment course [8, 9]. Thus, subclinical persistence of *Leishmania* after chemotherapeutic interventions could favor the development of acquired drug resistance and selection of non-susceptible parasite populations, risking the usefulness of available and potentially new drugs.

The systemic detection of subclinical *Leishmania* infection after clinical resolution of disease whether therapeutically achieved or after self-resolution, together with persistent asymptomatic infection, could reveal a previously unrecognized magnitude of the human population harboring viable parasites. The clinical and epidemiological impact of subclinical infection, and the factors that underlie parasite persistence or elimination, are unknown. In this study we explored clinical and parasitological factors associated with *Leishmania* persistence after standard-of-care treatment of cutaneous leishmaniasis (CL) caused by *L*. *Viannia*.

## Methods

### Ethics statement

This study was approved and monitored by the Institutional Review Board for Ethical Conduct of Research Involving Human Subjects of the Centro Internacional de Entrenamiento e Investigaciones Médicas (CIDEIM) with approval code CIEIH 1221, in accordance with national and international guidelines. All individuals voluntarily participated in the study. Written informed consent was obtained from each participant.

### Study design

This study was designed to explore the associations of clinical and parasitological factors with the persistence of *Leishmania* after supervised treatment and follow-up, and documented clinical resolution of CL. We included a cohort of CL patients who consulted CIDEIM outpatient clinics in Cali (Valle, Colombia) and Tumaco (Nariño, Colombia) between years 2011 and 2014, who participated in clinical studies that included prospective treatment follow-up. Demographic and clinical information from study participants was obtained at diagnosis and follow up visits.

The primary outcome of analysis was post-treatment parasite persistence. We qualitatively determined this by molecular detection of *Leishmania* nucleic acids (kDNA) from cutaneous lesions and nasal and tonsillar mucosa samples obtained from patients before and after treatment (end of treatment and at 13 weeks after initiation of treatment). Parasite viability was assessed by amplification and quantification of *Leishmania* 7SLRNA gene transcripts in all available lesion samples and for kDNA-positive mucosal samples [10]. Independent variables included sex, age, self-reported ethnicity, weight, self-reported time of lesion evolution, number of lesions, previous episode of leishmaniasis, and parasite loads in lesion aspirates, as well as drug-related variables including prescribed drug and adherence to treatment.

### Participants and samples

Clinical histories and samples from 70 adult patients with parasitological diagnosis of CL were included in this study. All participants were ≥18 years of age, received supervised standard-of-care treatment with meglumine antimoniate or miltefosine according to the national guidelines [11], and had clinical follow-up at the end of treatment and 13 weeks after initiation of treatment, the time at which therapeutic outcome was determined. Cure was defined as the re-epithelialization of all cutaneous lesions without inflammatory signs.

**Fig 1:**
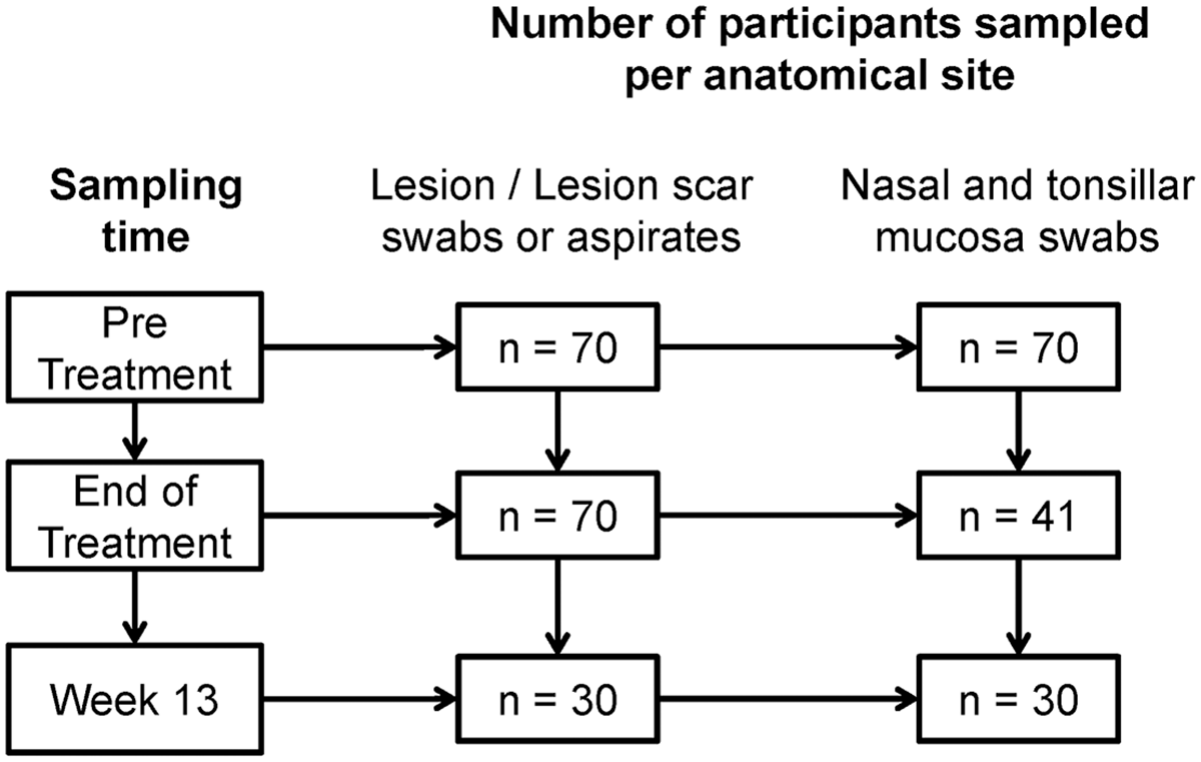
Schematic representation of study participants and samples obtained at different time points during treatment and clinical follow-up.

Samples of active lesions and lesion scars were taken by needle aspirate of the border of the lesion or at the periphery of the scar. Swab samples of cutaneous lesions, nasal mucosa, and palatine tonsil mucosa were obtained by gently rubbing sterile swabs (BuccalAmp kit; Epicenter Biotechnologies) over the mucosal surface [10]. Lesion and mucosal samples were collected from all patients before treatment, as were lesion samples at the end of treatment (Fig 1). Mucosal swabs were obtained from 41 participants at the end of treatment. Samples were also obtained from 30 patients at 13 weeks of follow-up. All samples were preserved in TRIzol Reagent (Life Technologies) and stored at -80°C until processed.

### Detection of *Leishmania*

DNA and RNA were extracted using the AllPrep DNA/RNA Minikit (Qiagen) according to manufacturer´s recommendations. *Leishmania* was detected by PCR amplification and southern blot of minicircle kDNA as previously described [4], which has a limit of detection of 0.3 fg of parasite DNA (~10^−2^ parasites per reaction) [12]. A positive control of *L*. *V*. *panamensis* (MHOM/CO/86/1166) DNA and a negative water control were included in each PCR run. PCR amplification of the human GAPDH gene was used as quality control of the extracted DNA [7]. DNAse treated RNA was used for quantitative reverse transcriptase PCR (qRT-PCR) of the *Leishmania* 7SLRNA transcript to evaluate parasite viability and quantify parasite burden. The limit of detection of this method is 10^2^ parasites per reaction [10]. Parasite loads were calculated by extrapolation to a standard curve and normalized to the number of human nucleated cells using TATA box binding protein amplification [7]. Detection of amplification products was performed using SYBR Green Master Mix (Applied Biosystems) on a BioRad CFX-96 platform. For those kDNA positive samples that were below the limit of detection of the 7SLRNA qRT-PCR, a maximum likelihood estimate of 0.0001 parasites per reaction was calculated as previously described [7].

### kDNA genotyping

A nested PCR reaction to amplify and sequence the conserved region of *Leishmania* minicircle kDNA was performed using external primers LVp1-Fw and LVp1-Rv and internal primers LVp1-Fw and LVp5-Rv [7]. Sequences were analyzed using BioEdit v7.2.5. Genetic distances and trees were calculated and constructed using MEGA 7.0. Population structure was explored with the STRUCTURE 2.3.4 software [13]. Runs were performed under the following parameters: burn-in period of 20,000 iterations, 200,000 Markov Chain Monte Carlo iterations and admixture model. A series of three runs was performed for each K value between 1 and 10. STRUCTURE outputs were visualized using STRUCTURE HARVESTER and used for selection of the number of genetic groups that best fitted the data [14]. Estimated fixation index (F_ST_) values were retrieved from STRUCTURE.

### Multilocus microsatellite typing (MLMT)

Fourteen microsatellite loci distributed in 13 *Leishmania* chromosomes were amplified by PCR from log-phase promastigote DNA [15]. The size of the microsatellites was determined by mobility of the PCR products in 4.5% agarose gels. Genetic distances were estimated using MSA4.05 and Populations-2.1 software. UPGMA trees were constructed using MEGA7.0 and compared to those generated by kDNA genotyping.

### Statistical analyses

Descriptive statistics were used to summarize the demographic, clinical and parasitological characteristics of the sample. Differences in frequencies were explored using McNemar’s test for paired nominal data, and X^2^ and Fisher's exact tests for unpaired data. Logistic regression analysis was used to identify independent predictors of post-treatment parasite persistence at the lesion site [16]. All variables associated with parasite persistence at the p<0.2 level in unadjusted analyses were entered into a multivariable model. The strength of association was determined by calculating the odds ratio (OR) and 95% confidence interval (CI) using the Wolf method [16, 17]. For the multivariable model, model fit using the goodness-of-fit test and model discrimination using the c-statistic, were analyzed [17]. All statistical analyses were performed using STATA 12 (StataCorp, College Station, TX, USA).

## Results

### Patient characteristics

Seventy patients with active CL, who received standard-of-care treatment with parenteral meglumine antimoniate (n = 38) or oral miltefosine (n = 32), were included in this study. Characteristics of the sample are summarized in Table 1. Patients were mostly young adult males of Afrocolombian or mestizo ethnicity. For the majority of participants (86%), the diagnosis was the first episode of CL. Clinical manifestations were predominantly characterized by single lesions with a median evolution time of 2.5 months. *L*.*V*. *panamensis* was the most frequently isolated species (in 93% of patients). Adherence to greater than 90% of the treatment regimen was achieved in 91% of study participants. None of the patients had symptoms or clinical signs of mucosal involvement.

**Table 1:** Clinical and demographic characteristics in patients with and without evidence of *Leishmania* persistence at the lesion site after clinical cure.

Variable	Total n = 70	kDNA / 7SLRNA^a^		*p*^b^
		Positive n = 32	Undetectable n = 33	
Sex, No. (%)				
Male	47 (67)	24 (56)	19 (44)	0.138*
Female	23 (33)	8 (36)	14 (64)	
Age, median (range), years	31 (18–72)	30(18–72)	31(20–65)	0.762**
Ethnicity, No. (%)				
Afrocolombian	50 (71)	23 (49)	24 (51)	0.939*
Other	20 (29)	9 (50)	9 (50)	
Municipality of infection No.(%)				
Nariño	46 (66)	18 (42)	25 (58)	0.097*
Other	24 (34)	14 (64)	8 (36)	
Weight, median (range), Kg	70 (11)	66 (48–102)	69 (50–94)	0.328**
Time of oldest lesion evolution, median (range), months	2.5 (1–40)	2(1–24)	2(1–40)	0.481**
Lesions per patient, median (range)	1 (1–8)	1 (1–8)	1 (1–6)	0.217**
Previous episode of leishmaniasis, No. (%)				
No	60 (86)	30 (55)	25 (45)	0.082^¶^
Yes	10 (14)	2 (20)	8 (80)	
Treatment, No. (%)				
Meglumine antimoniate	38 (46)	20 (59)	14 (41)	0.105*
Miltefosine	32 (54)	12 (39)	19 (61)	
Adherence to meglumine antimoniate (% ampules received/prescribed), median (range)	100 (50–100)	100 (50–100)	100 (70–100)	0.616**
Adherence to miltefosine (% capsules received/prescribed), median (range)	100 (76–100)	100 (76–100)	100 (88–100)	0.626**

^a^ Patients from whom end of treatment lesion samples were analyzed for *Leishmania* persistence. Samples from five patients were unavailable for molecular analyses.

^b^ Contrasts between samples from patients with and without molecular evidence of parasite persistence;

* X^2^ test;

** Mann-Whitney U test;

^¶^ 2-sided Fisher's exact test.

### *Leishmania* persistence after clinical cure of CL

**Fig 2:**
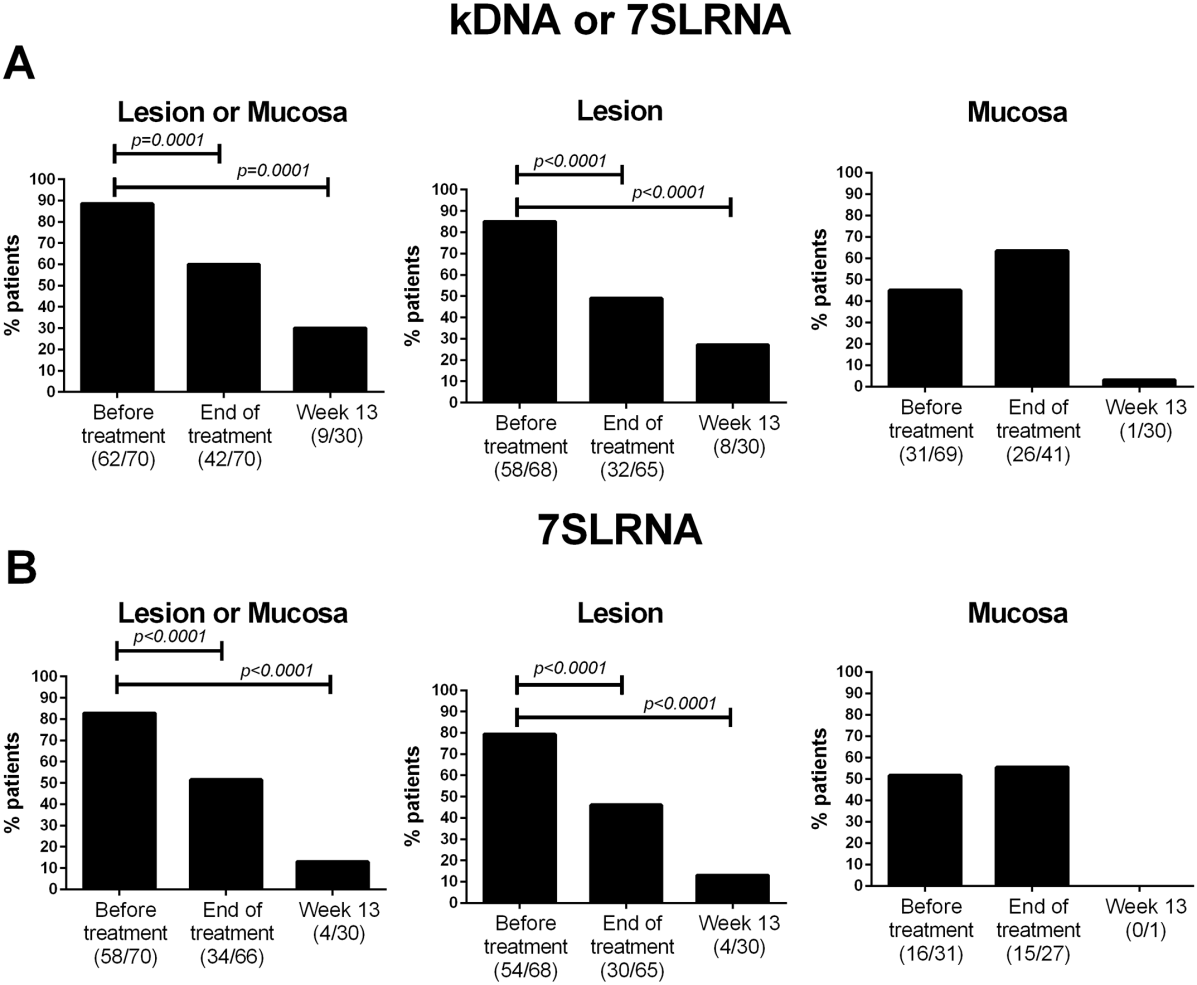
Frequency of detection of *Leishmania* in samples from CL patients before and after treatment. Presence of *Leishmania* was determined by detection of *Leishmania* kDNA or 7SLRNA in lesion and mucosal samples (A) and parasite viability (B) was determined by detection of 7SLRNA transcripts. Graphs represent frequency of positivity in at least one sample (left panels), or independently in lesions (center panels) and in any mucosal tissue (right panels). Data are shown as relative frequencies based on the total number of patients at each sampling time. Differences were analyzed by the McNemar’s test.

Evidence of *Leishmania* persistence (defined by amplification of minicircle kDNA or 7SLRNA transcripts in at least one lesion or mucosal sample) was found in 60% of patients (42/70) at the end of treatment and in 30% (9/30) at 13 weeks follow-up. Both of these proportions were significantly lower than the baseline parasite detection rate of 88% (62/70) before treatment (Fig 2A, left panel). Molecular detection of *Leishmania* in lesions was achieved in 49% of patients at the end of treatment and in 27% at 13 weeks, compared with 85% before treatment (Fig 2A, center panel). *Leishmania* was found in swab samples from mucosa in 45% of patients before treatment (Fig 2A, right panel). In contrast to the decline in the proportion of detection of parasites at the lesion site, no significant decrease in the frequency of patients with *Leishmania*-positive mucosal samples was found at the end of treatment compared with pre-treatment samples. However, only one of 30 patients had a positive mucosal sample at 13 weeks follow-up.

Parasite viability was evidenced by detection of 7SLRNA transcripts in 83% of patients (58/70) before treatment, indicating underestimation of the proportion of patients in which viable parasites were detected. This proportion decreased to 51% at the end of treatment and to 13% at 13 week of follow-up (Fig 2B, left panel). At the end of treatment, viable *Leishmania* were detected in a similar proportion of patients’ lesion and mucosal samples (46% and 55%, respectively; Fig 2B, center and right panels). At week 13, 7SLRNA transcripts were found in samples from lesions of only four patients. Parasite loads at the lesion site were overall lower at the end of treatment compared with parasite loads quantified before treatment (median parasite loads 14.5 and 40 parasites/1000 mammalian cells, respectively).

### Factors related with *Leishmania* persistence at the lesion site after clinical cure

Among the clinical and parasitological factors analyzed (Table 1), individuals with a prior history of CL were less likely to have detectable *Leishmania* after treatment and clinical cure than those without a previous episode of CL (20% vs. 55%, respectively). Adjusting for sex, the received drug and the municipality of infection (which were significant at *p<0*.*2*), a previous symptomatic episode of leishmaniasis was significantly associated with decreased risk of parasite persistence, with an adjusted odds ratio of 0.16 (95% CI: 0.03–0.92). The model fit well and discrimination was good (Table 2, c-statistic = 0.73).

**Table 2:** Multivariable logistic regression analysis of factors related to *Leishmania* parasite persistence at the lesion site at the end of the treatment.

Variable	Unadjusted odds ratio (95% CI)	Adjusted odds ratio (95% CI)
Male sex	2.21 (0.77–6.36)	2.02 (0.61–6.71)
Previous episode of leishmaniasis	0.21 (0.04–1.07)	0.16 (0.03–0.92)
Treatment with meglumine antimoniate	2.26 (0.84–6.11)	2.18 (0.70–6.85)
Municipality of infection (Nariño vs. Other)	0.41 (0.14–1.19)	0.37 (0.12–1.16)

Note: The final model included 65 patients; Goodness-of-Fit Test *p* = 0.62; c-statistic = 0.73.

### Genotyping of persistent parasite populations after treatment and clinical resolution of CL

**Fig 3:**
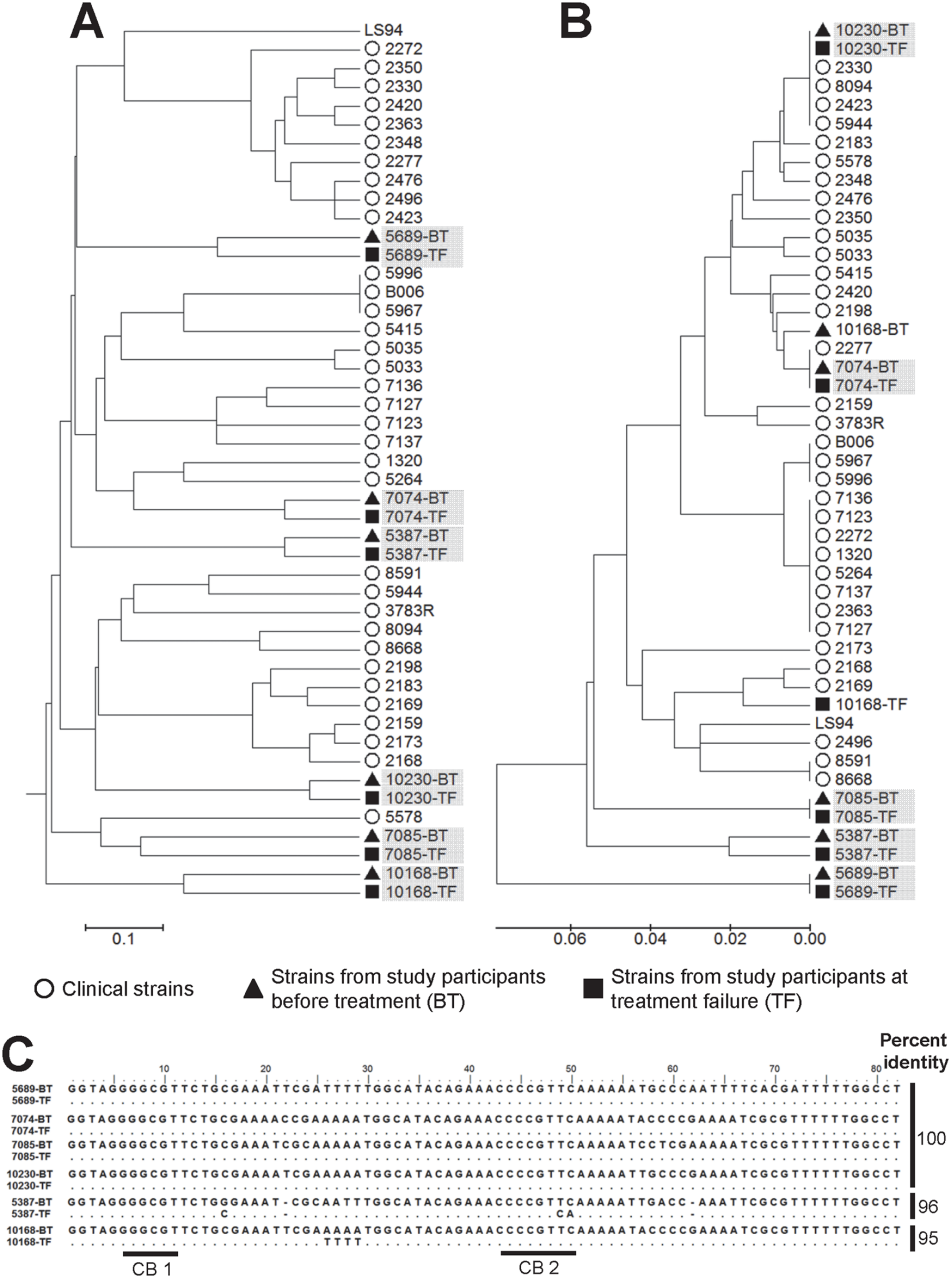
Comparative analysis of pre-treatment and post-treatment clinical strains by kDNA genotyping and multilocus microsatellite typing. UPGMA trees of distances calculated from MLMT data (A) and conserved block minicircle kDNA genotyping (B) from three pairs of *L*. *V*. *panamensis* and three pairs of *L*. *V*. *braziliensis* strains isolated before treatment (BT; black triangles) and at treatment failure (TF; black squares), alongside 34 *L*. *V*. *panamensis* clinical strains (open circles) and the reference *L*. *V*. *panamensis* strain LS94. Shadowed codes denote pairs of strains isolated from the same patient. (C) Pairwise sequence alignment of pre and post-treatment strains indicating % base-pair identity.

We have reported the use of genotyping of the conserved region of minicircle kDNA to explore the genetic diversity of *Leishmania* strains causing subclinical and asymptomatic infections [7]. To examine the relatedness of *Leishmania* populations during active disease and those that persist after treatment, we conducted an initial comparative analysis of multilocus microsatellite typing (MLMT) and kDNA genotyping of strains isolated before treatment and at treatment failure (S1 Table) from six CL patients. MLMT showed that parasites isolated before treatment grouped within the same branch as those isolated at treatment failure for each individual patient (Fig 3A, S2 Table). For five out of the six analyzed pairs of strains, groups defined by kDNA genotyping (Fig 3B, S3 Table) were concordant with those obtained by MLMT. Strains that were concordantly grouped by kDNA genotyping and MLMT shared ≥96% sequence homology in the sequenced region of the conserved block of minicircle kDNA (Fig 3C).

**Fig 4:**
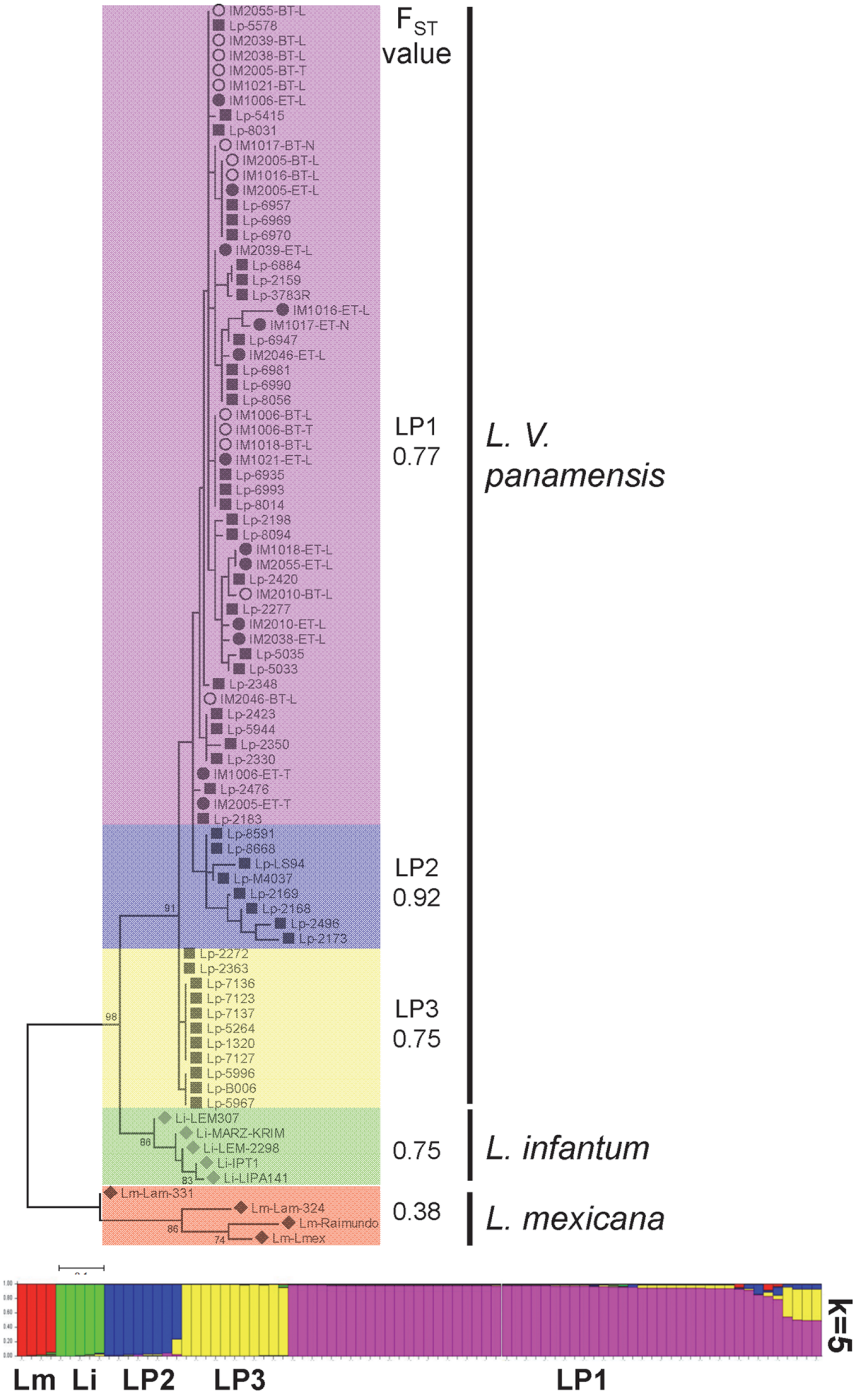
Analysis of *Leishmania* genetic diversity in clinical samples obtained from patients before and at the end of treatment. Maximum Likelihood tree of distances calculated from sequences of the conserved block of minicircle kDNA from lesion (-L), tonsillar (-T) and nasal (-N) mucosa samples obtained before treatment (BT) and at the end of treatment (ET), alongside a panel of *L*. *V*. *panamensis* (Lp; n = 48), *L*. *infantum* (Li; n = 5), and *L*. *mexicana* complex (Lm; n = 4) sequences. Color blocks denote subpopulations defined by population structure analysis and include the estimated fixation index (F_ST_). LP1-3: *L*. *V*. *panamensis* subpopulations. The bottom panel is the visual representation of the STRUCTURE analysis. Each strain/sequence (n = 83) is represented by a single vertical line divided into K colors, where K is the number of populations assumed (K = 5). Details of strains and kDNA sequences retrieved from NCBI are shown in S1 Table.

We performed kDNA genotyping on paired samples obtained pre-treatment and at the end of treatment from mucosal tissues (nasal and tonsillar) and lesions of 21 patients. Good quality sequences were obtained from 11 of the 21 patients: 10 sample pairs from lesion aspirates, two from tonsillar swabs, and one from nasal swabs (S3 Table). Genetic distances were calculated and population structure analyses were conducted alongside a panel of 46 *L*.*V*. *panamensis* clinical isolates obtained from CL patients across Colombia (Nariño [n = 31], Valle del Cauca [n = 5], Risaralda [n = 3], Choco [n = 6] and Putumayo [n = 1]), one *L*.*V*. *panamensis* reference strain, and one *L*.*V*. *panamensis*, 5 *L*. *infantum* and 4 *L*. *mexicana* kDNA sequences retrieved from NCBI (S1 Table). The selection of *L*.*V*. *panamensis* strains included in this panel and their proportions were reflective of the geographic distribution (municipality of infection) of study participants. Within the *L*.*V*. *panamensis* cluster, three subpopulations could be discerned responding primarily to geographic distribution: LP1 and LP2 corresponding to strains predominantly isolated from Nariño and LP3 to strains from Valle del Cauca and Risaralda. All minicircle kDNA sequences obtained from study participants pre- and post-treatment grouped within the LP1 cluster and fixation indices >0.3 supported the identified clusters (Fig 4). These data indicate that persistent *Leishmania* subpopulations within this group of CL patients were not genetically distinct from those isolated at diagnosis.

## Discussion

The outcome of antileishmanial chemotherapy is determined by clinical parameters of healing and non-healing responses. Although the parasitological response in spleen or bone marrow aspirates is a secondary indicator of therapeutic responsiveness during treatment of visceral leishmaniasis, microbiological clearance is not considered a reliable measure of healing of dermal disease [18, 19]. Our results revealed a high frequency of *Leishmania* persistence in mucosal tissues and at the lesion site in patients who were systematically followed 13 weeks after end of treatment, had a median overall treatment adherence of 100% and achieved therapeutic clinical cure of CL. Detection of kDNA molecules does not demonstrate parasite viability despite being rapidly degraded after parasite death [20]. However, due to the short half-life and lability of RNA molecules, detection of *Leishmania* 7SLRNA gene transcripts substantiates the persistence of viable parasites after clinical cure [10]. Although our current and previous findings [21] and that of others [22] show that clinical resolution of CL is accompanied by a reduction in parasite burden at the lesion site, *Leishmania* persistence is the norm rather than the exception, suggesting that other factors beyond parasite elimination contribute to the efficacy of antileishmanial therapy. In contrast to the significant reduction in the frequency of *Leishmania*-positive lesion samples, no decrease in the frequency of parasite detection at mucosal sites (either tonsillar or nasal mucosal samples) was found at the end of treatment. This observation suggests that mucosal sites could be a privileged niche for parasite persistence after drug treatment, either by pharmacokinetic differences in drug distribution and accumulation and/or immunological divergence between mucosal vs. skin tissues. Indeed, the detection of *Leishmania* in mucosal tissues in asymptomatic individuals and in CL patients without signs or symptoms of mucosal involvement [2, 7], and the development of mucosal disease years after an episode of CL, support the silent persistence of *Leishmania* in these anatomical sites.

Whether antileishmanial therapy should be aimed at complete parasite elimination (sterile cure) is a matter of debate. Persistent infection without signs of disease in animal models has provided evidence that such infection may promote immunity to subsequent infections [23–25]. In contrast, well documented clinical cases of disease reactivation in the context of immune suppression [26–28] or local trauma [29, 30] and mucosal involvement years after an episode of CL [31–33] support parasite persistence as a risk factor for reactivation of disease. Notably, the presence of a scar typical of CL and/or a positive Montenegro skin test reaction, both indicative of prior infection, have been shown to significantly increase the risk of re-activation of infection and development of CL in a prospective investigation of incidence of infection and disease in endemically exposed communities [33]. We have previously shown that amastigotes were less frequently observable in biopsies of active lesions of patients having scars suggestive of prior leishmaniasis [34]. Concordantly, our present results demonstrate a significant association between history of a previous episode of CL and a lower frequency of detectable *Leishmania* persistence at the end of treatment in CL patients who received supervised standard-of-care treatment with meglumine antimoniate or miltefosine. Interestingly, a negative skin test at diagnosis has been identified as a risk factor for relapse after treatment with pentavalent antimonials [35]. Therefore, it is plausible that acquired protective immune responses could contribute to enhanced parasite control during therapeutic intervention, thereby reducing the parasite burden, detectable parasite persistence and treatment failure.

Although not statistically significant, *Leishmania* persistence was more frequently detected in men, during treatment with meglumine antimoniate and among individuals from municipalities other than Nariño. In the case of parasitic infectious diseases, sexually mature men are often more susceptible to infection due to hormonal factors [36–38]. Higher susceptibility of male hamsters to *L*.*V*. *panamensis* infection has been associated with a more permissive immune environment for parasite survival [39]. However, the extent and causality of the relationship between sex and persistent infection after drug exposure in humans remains unknown. Pharmacokinetic and pharmacodynamic differences between drugs could also influence the post treatment persistence and burden of infection. Meglumine antimoniate has a short elimination half-life (~20h, [40]), in contrast to miltefosine which has a first elimination half-life of 7.05 days and a terminal elimination half-life of 30.9 days [41]. The short half-life of antimonials could result in reduced time of drug exposure of intracellular parasites promoting the higher frequency of parasite persistence. In addition, based on a retrospective cohort study of 230 CL patients, we have recently found that treatment with meglumine antimoniate (vs. miltefosine) is a risk factor for therapeutic failure [42]. The potential contribution of other demographic variables to *Leishmania* persistence such as genetic background or race remains to be determined. However, that individuals from municipalities other than Nariño presented higher frequency of parasite persistence could be indicative of parasite subpopulations or host determinants related to geographic origin, specifically contributing to *Leishmania* persistence.

Selection of parasite subpopulations within the human host is considered to contribute to emergence of drug resistance. Characterization of parasites causing subclinical infections is restricted by the inability to isolate, culture, and propagate the parasite. Based on kDNA sequence homology and population structure analyses, strains and patient samples could be clustered based on geographical origin. However, we found no evidence of selection of parasite subpopulation after treatment and clinical cure. Phenotypic analyses such as drug susceptibility testing, and sequence analyses of kDNA and ITS rDNA have revealed differences in parasites isolated at diagnosis and at treatment failure in individuals with cutaneous and visceral leishmaniasis [8, 9, 43, 44]. It is plausible that implementation of sequencing methods at the genome level could contribute to discriminate and characterize subclinically persistent *Leishmania* subpopulations.

Association analyses of clinical, parasitological and drug-related factors with parasite persistence at later time points (eg. week 13 and 26 after end of treatment) were precluded in this study due to sample size limitations. However, our results and those of others demonstrate that currently available treatments do not eliminate *Leishmania*. This contributes to the proportion of the persistently infected human population, which includes patients with therapeutically achieved cure, those with self-resolution of active disease and individuals with asymptomatic infection. Despite compelling circumstantial evidence, anthroponotic transmission of *Leishmania (Viannia)* remains uncertain. Nevertheless, the high proportion of individuals harboring infection in endemic areas, coupled with the rising importance of domestic transmission of CL, and the demonstrated risk of disease re-activation [45–47], highlight the importance of this subclinically infected human population in control strategies for CL. Prospective studies designed to interrogate the dynamics of parasite clearance and reactivation of disease are needed to establish the clinical and epidemiological impact of *Leishmania* persistence.

## Supporting information

S1 Table *Leishmania* strains and sequences. (DOCX)

S2 Table Multilocus microsatellite typing profiles of *L*. *Viannia* strains. (DOCX)

S3 Table Minicircle kDNA conserved block sequences from clinical samples and strains. (DOCX)
