# Supplementary material for: Clinical and parasitological factors in parasite persistence after treatment and clinical cure of cutaneous leishmaniasis
Source: PLoS Negl Trop Dis. 2017 Jul 13;11(7):e0005713. doi: 10.1371/journal.pntd.0005713 (PMC5526576; doi:10.1371/journal.pntd.0005713)
Supplement: S1 Table — (DOCX) [file pntd.0005713.s001.docx]

**Supporting Table 1. *Leishmania* strains and sequences**

| **GenBank SEQUENCES** | | |
| --- | --- | --- |
| **Species** | **Strain name** | **GenBank Accesion Number** |
| *L.* (*L*)*. infantum* | MHOM/ES/81/LEM307 | AF188701.1 |
|  | MHOM/TN/80/IPT1 ^a^ | Z35274.1 |
|  | MHOM/FR/91/LEM-2298 | AF190475.1 |
|  | MHOM/DZ/85/LIPA141 | AF169133.1 |
|  | MHOM/SU/84/MARZ-KRIM | AF190476.1 |
| *L. mexicana* Complex | *L. amazonensis* Lam-331 | EU370875.1 |
|  | MHOM/BR/00/Raimundo | M21326.1 |
|  | *L. amazonensis* isolate Lam-324 | EU370871.1 |
|  | *L. mexicana* | Z11555.1 |
| *L.* (*V*)*. panamensis* | MHOM/PA/75/M4037 | AF118474.1 |
| *L.* (*V*)*. braziliensis* | MHOM/BR/75/M2904 | XM_001568753.1 |
| **CLINICAL STRAINS** | | |
| **Species** | **Strain name** |  |
| *L.* (*V*)*. braziliensis* | MHOM/CO/09/5689 (BT) ^b^ |  |
|  | MHOM/CO/10/5689 (FT) ^c^ |  |
|  | MHOM/CO/08/5387 (BT) |  |
|  | MHOM/CO/08/5387 (FT) |  |
|  | MHOM/CO/12/7085 (BT) |  |
|  | MHOM/CO/12/7085 (FT) |  |
| *L.* (*V*)*. panamensis* | MHOM/PA/1971/LS94 ^a^ |  |
|  | MHOM/CO/11/5996 |  |
|  | MHOM/CO/12/B006 |  |
|  | MHOM/CO/11/5967 |  |
|  | MHOM/CO/85/2277 |  |
|  | MHOM/CO/85/2476 |  |
|  | MHOM/CO/85/2496 |  |
|  | MHOM/CO/85/2423 |  |
|  | MHOM/CO/85/2272 |  |
|  | MHOM/CO/85/2350 |  |
|  | MHOM/CO/85/2330 |  |
|  | MHOM/CO/85/2420 |  |
|  | MHOM/CO/85/2363 |  |
|  | MHOM/CO/85/2348 |  |
|  | MHOM/CO/84/2198 |  |
|  | MHOM/CO/84/2159 |  |
|  | MHOM/CO/84/2173 |  |
|  | MHOM/CO/84/2168 |  |
|  | MHOM/CO/84/2183 |  |
|  | MHOM/CO/84/2169 |  |
|  | MHOM/CO/87/1320 |  |
|  | MHOM/CO/09/5578 |  |
|  | MHOM/CO/06/8591 |  |
|  | MHOM/CO/08/5415 |  |
|  | MHOM/CO/11/5944 |  |
|  | MHOM/CO/07/5264 |  |
|  | MHOM/CO/05/8094 |  |
|  | MHOM/CO/06/8668 |  |
|  | MHOM/CO/05/5035 |  |
|  | MHOM/CO/05/5033 |  |
|  | MHOM/CO/03/3783 |  |
|  | MHOM/CO/12/7136 |  |
|  | MHOM/CO/12/7123 |  |
|  | MHOM/CO/13/7127 |  |
|  | MHOM/CO/12/7137 |  |
|  | MHOM/CO/04/6957 |  |
|  | MHOM/CO/04/6969 |  |
|  | MHOM/CO/04/6970 |  |
|  | MHOM/CO/04/6884 |  |
|  | MHOM/CO/04/6947 |  |
|  | MHOM/CO/04/6981 |  |
|  | MHOM/CO/04/6990 |  |
|  | MHOM/CO/04/8056 |  |
|  | MHOM/CO/04/6935 |  |
|  | MHOM/CO/04/6993 |  |
|  | MHOM/CO/04/8014 |  |
|  | MHOM/CO/04/8031 |  |
|  | MHOM/CO/12/7074 (BT) |  |
|  | MHOM/CO/13/7074 (TF) |  |
|  | MHOM/CO/09/10168 (BT) |  |
|  | MHOM/CO/09/10168 (TF) |  |
|  | MHOM/CO/09/10230 (BT) |  |
|  | MHOM/CO/10/10230 (TF) |  |

^a^ Reference strains

^b^ Strains isolated at diagnosis (BT: before treatment)

^c^ Strains isolated at treatment failure (TF: treatment failure)
