# Supplementary material for: Clinical and parasitological factors in parasite persistence after treatment and clinical cure of cutaneous leishmaniasis
Source: PLoS Negl Trop Dis. 2017 Jul 13;11(7):e0005713. doi: 10.1371/journal.pntd.0005713 (PMC5526576; doi:10.1371/journal.pntd.0005713)
Supplement: S3 Table — (DOCX) [file pntd.0005713.s003.docx]

**Supporting Table 3. Minicircle kDNA conserved block sequences from clinical samples and strains**

| **Sequence ID** | **Minicircle kDNA conserved block sequence: 5´🡪 3´** |
| --- | --- |
| IM1016-ET-L | GGTAGGGGCGTTCTGCGAAATCGCAAAAATGGCATACAGAAACCCCGTTCAAAAAATCCCGGAAAATCCCGTTTTTTGGCCT |
| IM1017-ET-N | GGTAGGGGCGTTCTGCGAAATCGCAAAAATGGCATACAGAAACCCCGTTCAGAAATACCCCAAAAATCGCGTTTTTTGGCCT |
| IM1006-BT-L | GGTAGGGGCGTTCTGCGAAAACCGAAAAATGGCATACAGAAACCCCGTTCAAAAATTCCCCGAAAATCGCGTTTTTTGGCCT |
| IM1006-ET-L | GGTAGGGGCGTTCTGCGAAAATCGAAAAATGGCATACAGAAACCCCGTTCAAAAATTCCCCGAAAATCGCGTTTTTTGGCCT |
| IM1006-BT-T | GGTAGGGGCGTTCTGCGAAAACCGAAAAATGGCATACAGAAACCCCGTTCAAAAATTCCCCGAAAATCGCGTTTTTTGGCCT |
| IM1006-ET-T | GGTAGGGGCGTTCTGCGAAAATCGAAAAATGGCATACAGAAACCCCGTTCAAAAAATGCCCGAAAATCGCGTTTTTTGGCCT |
| IM1016-BT-L | GGTAGGGGCGTTCTGCGAAATTCGAAAAATGGCATACAGAAACCCCGTTCAAAAATTGCCCGAAAATCGCGTTTTTTGGCCT |
| IM1017-BT-N | GGTAGGGGCGTTCTGCGAAATTCGAAAAATGGCATACAGAAACCCCGTTCAAAAATTCCCCGAAAATCGCGTTTTTTGGCCT |
| IM1018-BT-L | GGTAGGGGCGTTCTGCGAAAACCGAAAAATGGCATACAGAAACCCCGTTCAAAAATACCCCGAAAATCGCGTTTTTTGGCCT |
| IM1018-ET-L | GGTAGGGGCGTTCTGCGAAAATCGAAAAATGGCATACAGAAACCCCGTTCAAAAATCCCCCAAAATTCGCGTTTTTTGGCCT |
| IM1021-BT-L | GGTAGGGGCGTTCTGCGAAAATCGAAAAATGGCATACAGAAACCCCGTTCAAAAATTCCCCGAAAATCGCGTTTTTTGGCCT |
| IM1021-ET-L | GGTAGGGGCGTTCTGCGAAAACCGAAAAATGGCATACAGAAACCCCGTTCAAAAATTCCCCGAAAATCGCGTTTTTTGGCCT |
| IM2005-BT-L | GGTAGGGGCGTTCTGCGAAATTCGAAAAATGGCATACAGAAACCCCGTTCAAAAATTGCCCGAAAATCGCGTTTTTTGGCCT |
| IM2005-ET-L | GGTAGGGGCGTTCTGCGAAATTCGAAAAATGGCATACAGAAACCCCGTTCAAAAATTGCCCGAAAATCGCGTTTTTTGGCCT |
| IM2005-BT-T | GGTAGGGGCGTTCTGCGAAAATCGAAAAATGGCATACAGAAACCCCGTTCAAAAATACCCCGAAAATCGCGTTTTTTGGCCT |
| IM2005-ET-T | GGTAGGGGCGTTCTGCGAAAATCGAAAAATGGCATACAGAAACCCCGTTCAAAAAATGCCCGAAAATCGCGTTTTTTGGCCT |
| IM2010-BT-L | GGTAGGGGCGTTCTGCGAAATCCGAAAAATGGCATACAGAAACCCCGTTCAAAAATCCCCCAAAATTCGCGTTTTTTGGCCT |
| IM2010-ET-L | GGTAGGGGCGTTCTGCGAAAACCGAAAAATGGCATACAGAAACCCCGTTCAAAAATCCTCGAAAAATCGCGTTTTTTGGCCT |
| IM2038-BT-L | GGTAGGGGCGTTCTGCGAAAATCGAAAAATGGCATACAGAAACCCCGTTCAAAAATTCCCCGAAAATCGCGTTTTTTGGCCT |
| IM2038-ET-L | GGTAGGGGCGTTCTGCGAAATCCGAAAAATGGCATACAGAAACCCCGTTCAAAAATCCCCCAAAAATCGCGTTTTTTGGCCT |
| IM2039-BT-L | GGTAGGGGCGTTCTGCGAAAATCGAAAAATGGCATACAGAAACCCCGTTCAAAAATTCCCCGAAAATCGCGTTTTTTGGCCT |
| IM2039-ET-L | GGTAGGGGCGTTCTGCGAAAATGGAAAAATGGCATACAGAAACCCCGTTCAAAAATTCCCCGAAAATCGCGTTTTTTGGCCT |
| IM2046-BT-L | GGTAGGGGCGTTCTGCGAAAATCGAAAAATGGCATACAGAAACCCCGTTCAAAAATTGCCCGAAAATCGCGTTTTTTGGCCT |
| IM2046-ET-L | GGTAGGGGCGTTCTGCGAAATTGGAAAAATGGCATACAGAAACCCCGTTCAAAAATTGCCCGAAAATCGCGTTTTTTGGCCT |
| IM2055-BT-L | GGTAGGGGCGTTCTGCGAAAATCGAAAAATGGCATACAGAAACCCCGTTCAAAAATTCCCCGAAAATCGCGTTTTTTGGCCT |
| IM2055-ET-L | GGTAGGGGCGTTCTGCGAAAATCGAAAAATGGCATACAGAAACCCCGTTCAAAAATCCCCCAAAATTCGCGTTTTTTGGCCT |
| Lp-LS94 | GGTAGGGGCGTTCTGCGAAAATCGTTTTTTGGCATACAGAAACCCCGTTCAAAAAATACCCGAAAATCACGTTTTTTGGCCT |
| Lp-M4037 | GGTAGGGGCGTTCTGCGAAAATCGAATTTTGGCATACAGAAACCCCGTTCAAAAAATGCCCGAAAATCACGTTTTTTGGCCT |
| Lp-2423 | GGTAGGGGCGTTCTGCGAAAACCGAAAAATGGCATACAGAAACCCCGTTCAAAAATTGCCCGAAAATCGCGTTTTTTGGCCT |
| Lp-5944 | GGTAGGGGCGTTCTGCGAAAACCGAAAA-TGGCATACAGAAACCCCGTTCAAAAATTGCCCGAAAATCGCGTTTTTTGGCCT |
| Lp-7136 | GGTAGGGGCGTTCTGCGAAAACCGAAAAATGGCATACAGAAACCCCGTTCAAAAAATGCCCGAAAATCGCTATTTTTGGCCT |
| Lp-7123 | GGTAGGGGCGTTCTGCGAAAACCGAAAAATGGCATACAGAAACCCCGTTCAAAAAATGCCCGAAAATCGCTATTTTTGGCCT |
| Lp-7137 | GGTAGGGGCGTTCTGCGAAAACCGAAAAATGGCATACAGAAACCCCGTTCAAAAAATGCCCGAAAATCGCTATTTTTGGCCT |
| Lp-5996 | GGTAGGGGCGTTCTGCGAAATTCGAAAAATGGCATACAGAAACCCCGTTCAAAAAATGGCCGAAAATCGCTATTTTTGGCCT |
| Lp-B006 | GGTAGGGGCGTTCTGCGAAATTCGAAAAATGGCATACAGAAACCCCGTTCAAAAAATG-CCGAAAATCGCTATTTTTGGCCT |
| Lp-5967 | GGTAGGGGCGTTCTGCGAAATTCGAAAAATGGCATACAGAAACCCCGTTCAAAAAATGCCCGAAAATCGCTATTTTTGGCCT |
| Lp-5578 | GGTAGGGGCGTTCTGCGAAAATCGAAAAATGGCATACAGAAACCCCGTTCAAAAATTCCCCGAAAATCGCGTTTTTTGGCCT |
| Lp-2272 | GGTAGGGGCGTTCTGCGAAAATCGAAAAATGGCATACAGAAACCCCGTTCAAAAAATGCCCGAAAATCGCTATTTTTGGCCT |
| Lp-8591 | GGTAGGGGCGTTCTGCGAAAATCGAATTTTGGCATACAGAAACCCCGTTCAAAAAATGCCCGAAAATCGCGTTTTTTGGCCT |
| Lp-5415 | GGTAGGGGCGTTCTGCGAAAATCGAAAAATGGCATACAGAAACCCCGTTCAAAAATACCCCGAAAATCACGTTTTTTGGCCT |
| Lp-2198 | GGTAGGGGCGTTCTGCGAAAATCGACAAATGGCATACAGAAACCCCGTTCAAAAATACCCCAAAAATCGCGTTTTTTGGCCT |
| Lp-8668 | GGTAGGGGCGTTCTGCGAAAATCGAATTTTGGCATACAGAAACCCCGTTCAAAAAATGCCCGAAAATCGCGTTTTTTGGCCT |
| Lp-2168 | GGTAGGGGCGTTCTGCGAAAATCGATTTTTGGCATACAGAAACCCCGTTCAAAAATACCCCAAAATTCGCGTTTTTTGGCCT |
| Lp-5035 | GGTAGGGGCGTTCTGCGAAAACCGAAAAATGGCATACAGAAACCCCGTTCAAAAATTCCCCAAAATTCGCCTTTTTTGGCCT |
| Lp-5033 | GGTAGGGGCGTTCTGCGAAAACCGAAAAATGGCATACAGAAACCCCGTTCAAAAATTCCCCAAAAATCGCCTTTTTTGGCCT |
| Lp-2350 | GGTAGGGGCGTTCTGCGAAAACCTTAAAATGGCATACAGAAACCCCGTTCAAAAATTGCCCGAAAATCGCGTTTTTTGGCCT |
| Lp-2420 | GGTAGGGGCGTTCTGCGAAAACCGAAAAATGGCATACAGAAACCCCGTTCAAAAATACCCCAAAATTCGCGTTTTTTGGCCT |
| Lp-2363 | GGTAGGGGCGTTCTGCGAAAATCGAAAAATGGCATACAGAAACCCCGTTCAAAAAATGCCCGAAAATCGCTATTTTTGGCCT |
| Lp-2183 | GGTAGGGGCGTTCTGCGAAAATCGAAAAATGGCATACAGAAACCCCGTTCAAAAAATGCCCGAAAATCGCGTTTTTTGGCCT |
| Lp-2169 | GGTAGGGGCGTTCTGCGAAAATCGATTTTTGGCATACAGAAACCCCGTTCAAAAAAACCCCGAAATTCGCGTTTTTTGGCCT |
| Lp-3783R | GGTAGGGGCGTTCTGCGAAAATGCAAAAATGGCATACAGAAACCCCGTTCAAAAATAACCCGAAAATCGCGTTTTTTGGCCT |
| Lp-2348 | GGTAGGGGCGTTCTGCGAAAATCGAAAAATGGCATACAGAAACCCCGTTCAAAAATTACCCGAAAATCGCGTTTTTTGGCCT |
| Lp-2476 | GGTAGGGGCGTTCTGCGAAATTCGAAAAATGGCATACAGAAACCCCGTTCAAAAAATGCCCGAAAATCGCGTTTTTTGGCCT |
| Lp-2496 | GGTAGGGGCGTTTTGCGAAAATCGATTTTTGGCATACAGAAACCCCGTTCAAAAATTACCCGAAAATCGCGTTTTTTGGCCT |
| Lp-5264 | GGTAGGGGCGTTCTGCGAAAACCGAAAAATGGCATACAGAAACCCCGTTCAAAAAATGCCCGAAAATCGCTATTTTTGGCCT |
| Lp-1320 | GGTAGGGGCGTTCTGCGAAAACCGAAAAATGGCATACAGAAACCCCGTTCAAAAAATGCCCGAAAATCGCTATTTTTGGCCT |
| Lp-7127 | GGTAGGGGCGTTCTGCGAAAACCGAAAAATGGCATACAGAAACCCCGTTCAAAAAATGCCCGAAAATCGCTATTTTTGGCCT |
| Lp-2277 | GGTAGGGGCGTTCTGCGAAAACCGAAAAATGGCATACAGAAACCCCGTTCAAAAATACCCCAAAAaTCGCGTTTTTTGGCCT |
| Lp-2330 | GGTAGGGGCGTTCTGCGAAAACCGAAAAATGGCATACAGAAACCCCGTTCAAAAATTGCCCGAAAATCGCGTTTTTTGGCCT |
| Lp-8094 | GGTAGGGGCGTTCTGCGAAAATCGAAAAATGGCATACAGAAACCCCGTTCAAAAATTGCCCAAAAATCGCGTTTTTTGGCCT |
| Lp-6957 | GGTAGGGGCGTTCTGCGAAATTCGAAAAATGGCATACAGAAACCCCGTTCAAAAATTGCCCGAAAATCGCGTTTTTTGGCCT |
| Lp-6969 | GGTAGGGGCGTTCTGCGAAATTCGAAAAATGGCATACAGAAACCCCGTTCAAAAATTGCCCGAAAATCGCGTTTTTTGGCCT |
| Lp-6970 | GGTAGGGGCGTTCTGCGAAATTCGAAAAATGGCATACAGAAACCCCGTTCAAAAATTGCCCGAAAATCGCGTTTTTTGGCCT |
| Lp-6884 | GGTAGGGGCGTTCTGCGAAAATGCAAAAATGGCATACAGAAACCCCGTTCAAAAATAGCCCGAAAATCGCGTTTTTTGGCCT |
| Lp-6947 | GGTAGGGGCGTTCTGCGAAATTGGAAAAATGGCATACAGAAACCCCGTTCAAAAATACCCCAAAAATCGCGTTTTTTGGCCT |
| Lp-6981 | GGTAGGGGCGTTCTGCGAAATTGGAAAAATGGCATACAGAAACCCCGTTCAAAAATACCCCGAAAATCGCGTTTTTTGGCCT |
| Lp-6990 | GGTAGGGGCGTTCTGCGAAATTGGAAAAATGGCATACAGAAACCCCGTTCAAAAATACCCCGAAAATCGCGTTTTTTGGCCT |
| Lp-8056 | GGTAGGGGCGTTCTGCGAAATTGGAAAAATGGCATACAGAAACCCCGTTCAAAAATACCCCGAAAATCGCGTTTTTTGGCCT |
| Lp-6935 | GGTAGGGGCGTTCTGCGAAAACCGAAAAATGGCATACAGAAACCCCGTTCAAAAATTCCCCGAAAATCGCGTTTTTTGGCCT |
| Lp-6993 | GGTAGGGGCGTTCTGCGAAAACCGAAAAATGGCATACAGAAACCCCGTTCAAAAATTCCCCGAAAATCGCGTTTTTTGGCCT |
| Lp-8014 | GGTAGGGGCGTTCTGCGAAAACCGAAAAATGGCATACAGAAACCCCGTTCAAAAATACCCCGAAAATCGCGTTTTTTGGCCT |
| Lp-8031 | GGTAGGGGCGTTCTGCGAAAATCGAAAAATGGCATACAGAAACCCCGTTCAAAAATTCCCCGAAAATCGCGTTTTTTGGCCT |
| Lp-2159 | GGTAGGGGCGTTCTGCGAAAATGCAAAAATGGCATACAGAAACCCCGTTCAAAAATTGCCCGAAAATCGCGTTTTTTGGCCT |
| Lp-2173 | GGTAGGGGCGTTTTGCGAAAATCGAATTTTGGCATACAGAAACCCCGTTCAAAAATTCCCCAAAAATCGCTATTTTTGGCCT |
| 5387-BT | GGTAGGGGCGTTCTGGGAAAT-CGCAATTTGGCATACAGAAACCCCGTTCAAAAATTGACC-AAATTCGCGTTTTTTGGCCT |
| 5387-TF | GGTAGGGGCGTTCTGCGAAAT-CGCAATTTGGCATACAGAAACCCCGTCAAAAAATTGACC-AAATTCGCGTTTTTTGGCCT |
| 5689-BT | GGTAGGGGCGTTCTGCGAAATTCGATTTTTGGCATACAGAAACCCCGTTCAAAAAATGCCCAATTTTCACGATTTTTGGCCT |
| 5689-TF | GGTAGGGGCGTTCTGCGAAATTCGATTTTTGGCATACAGAAACCCCGTTCAAAAAATGCCCAATTTTCACGATTTTTGGCCT |
| 7074-BT | GGTAGGGGCGTTCTGCGAAAACCGAAAAATGGCATACAGAAACCCCGTTCAAAAATACCCCGAAAATCGCGTTTTTTGGCCT |
| 7074-TF | GGTAGGGGCGTTCTGCGAAAACCGAAAAATGGCATACAGAAACCCCGTTCAAAAATACCCCGAAAATCGCGTTTTTTGGCCT |
| 7085-BT | GGTAGGGGCGTTCTGCGAAATCGCAAAAATGGCATACAGAAACCCCGTTCAAAAATCCTCGAAAAATCGCGTTTTTTGGCCT |
| 7085-TF | GGTAGGGGCGTTCTGCGAAATCGCAAAAATGGCATACAGAAACCCCGTTCAAAAATCCTCGAAAAATCGCGTTTTTTGGCCT |
| 10168-BT | GGTAGGGGCGTTCTGCGAAATTCGAAAAATGGCATACAGAAACCCCGTTCAAAAATACCCCGAAAATCGCGTTTTTTGGCCT |
| 10168-TF | GGTAGGGGCGTTCTGCGAAATTCGATTTTTGGCATACAGAAACCCCGTTCAAAAATACCCCGAAAATCGCGTTTTTTGGCCT |
| 10230-BT | GGTAGGGGCGTTCTGCGAAAATCGAAAAATGGCATACAGAAACCCCGTTCAAAAATTGCCCGAAAATCGCGTTTTTTGGCCT |
| 10230-TF | GGTAGGGGCGTTCTGCGAAAATCGAAAAATGGCATACAGAAACCCCGTTCAAAAATTGCCCGAAAATCGCGTTTTTTGGCCT |
| Li-IPT1 | GGTAGGGGCGTTCTGCGAAATCGGAAAAATGG-GTGCAGAAATCCCGTTCAAAAATCGACCAAAAAT-GCCAAAAATCGGCT |
| Li-LIPA141 | GGTAGGGGCGTTCTGCAAAATCGGAAAAATGG-GTGCAGAAATCCCGTTCAAAAATCGACCAAAAAT-GCCAAAAATCGGCT |
| Li-MARZ-KRIM | GGTAGGGGCGTTCTGCGAAAACCGAAAAATGG-GTGCAGAAATCCCGTTCAAAAATTGGCCGAAAAT-GCCAAAAATCGGCT |
| Li-LEM-2298 | GGTAGGGGCGTTCTGCGAAATCCGAAAAATGG-GTGCAGAAATCCCGTTCAAAAAT-GGCCGAAAAT-GCCAAAAATCGGCT |
| Li-LEM307 | GGTAGGGGCGTTCTGCGAAAATGGAAAAATGG-GTGCAGAAATCCCGTTCAAAAAAGGGCCGAAAAT-GCCAAAAATCGGCT |
| Lm-Lam-331 | GGTAGGGGCGTTCTGCGAAAATGGGAAAAATGAGTGCAGAAACCCCGTTCATAATTTGGCCAAAAATCCTGGAAATCGGCTC |
| Lm-Raimundo | GGGAGGGGCGTTCTGCGGAAACCTCAAAAATGAGTGCAGAAACCCCGTTCATATTTTGGGGGATTTTTGGGAATTTCGGTTC |
| Lm-Lam-324 | GGTAGGGGCGTTCTGCG-AAACGGGGAAAATGAGTGCAGAAACCCCGTTCATATTTTGGGGAATTTTGGCCGAAAATGCCTC |
| Lm-Lmex | GGGAGGGGCGTTCTGCGAATTTGGGAAAAATGAGTGCAGAAACCCCGTTCATATTTTGGGGAATTTTGGGGAATTCCGGCTC |
